# Supplementary material for: The Systems Biology Research Tool: evolvable open-source software
Source: BMC Syst Biol. 2008 Jun 29;2:55. doi: 10.1186/1752-0509-2-55 (PMC2446383; doi:10.1186/1752-0509-2-55)
Supplement: Additional file 1 — SBRT Archive. An archive of the current version of the Systems Biology Research Tool. [file 1752-0509-2-55-S1.zip › sbrt-1.4.0/doc/developers_guide/api/sbrt/shell/io/class-use/TextInputFile.html]

Uses of Class sbrt.shell.io.TextInputFile


|  |  |  |
| --- | --- | --- |
| **Overview** \| **Package** \| **Class** \| **Use** \| **Tree** \| **Deprecated** \| **Index** \| **Help** | |  |
| PREV   NEXT | **FRAMES**    **NO FRAMES**     **All Classes** |  |


---


## **Uses of Class sbrt.shell.io.TextInputFile**

| Packages that use TextInputFile | |
| --- | --- |
| **sbrt.shell.io** | Provides classes and interfaces for reading and writing files. |
| **sbrt.shell.io.fba** | Provides classes and interfaces for reading and writing files relevant to Flux Balance Analysis. |
| **sbrt.shell.mng** | Provides classes and interfaces for creating process managers. |
| **sbrt.shell.mng.fba** | Provides classes and interfaces for creating Flux Balance Analysis process managers. |

Uses of TextInputFile in sbrt.shell.io

| Methods in sbrt.shell.io that return TextInputFile | |
| --- | --- |
| `static TextInputFile<java.lang.String>` | `TextInputFile.getBasic(java.lang.String fileName)`             Returns a basic text input file. |
| `static TextInputFile<java.lang.String>` | `TextInputFile.getBasic(java.lang.String fileName, FileFormat fileFormat)`             Returns a basic text input file. |

Constructors in sbrt.shell.io with parameters of type TextInputFile

`TextFileLineIterator(TextInputFile<E> file)`
  
          Constructs a new text file line iterator using the
provided text input file.

Uses of TextInputFile in sbrt.shell.io.fba

| Subclasses of TextInputFile in sbrt.shell.io.fba | |
| --- | --- |
| `class` | `RxnNamExprSetInputFile<E extends MathExpr<S>,S>`             This class is used to read files containing a set of reaction name expressions. |

Uses of TextInputFile in sbrt.shell.mng

| Methods in sbrt.shell.mng that return TextInputFile | |
| --- | --- |
| `static TextInputFile<java.lang.String>` | `AbstractProcessManager.getFileNameSource(ManagerInput<java.lang.String,java.lang.String> input, java.io.PrintWriter out)`             Returns the parsed value for `AbstractProcessManager.OUTPUT_FILE_NAME_FILE` in the provided input and prints a descriptive message to the provided print writer. |

Uses of TextInputFile in sbrt.shell.mng.fba

| Methods in sbrt.shell.mng.fba that return TextInputFile | |
| --- | --- |
| `static TextInputFile<LinearComb<java.lang.String>>` | `ObjectiveFunctionManager.getInputFile(ManagerInput<java.lang.String,java.lang.String> input, java.io.PrintWriter out, Fluxome fluxome)`             Returns the file denoted by `ObjectiveFunctionManager.OBJ_FUNCT_FILE` in the provided manager input. |
| `static TextInputFile<LinearComb<java.lang.String>>` | `ObjectiveFunctionManager.getInputFile(java.lang.String fileName, FileFormat format, Fluxome fluxome)`             Returns the input file with the provided name. |
| `static TextInputFile<LinearComb<java.lang.String>>` | `ObjectiveFunctionManager.getInputFile(java.lang.String fileName, Fluxome fluxome)`             Returns the input file with the provided name. |

---


|  |  |  |
| --- | --- | --- |
| **Overview** \| **Package** \| **Class** \| **Use** \| **Tree** \| **Deprecated** \| **Index** \| **Help** | |  |
| PREV   NEXT | **FRAMES**    **NO FRAMES**     **All Classes** |  |


---
